# Mechanisms of arrhythmogenesis related to calcium-driven alternans in a model of human atrial fibrillation

**DOI:** 10.1038/srep36395

**Published:** 2016-11-04

**Authors:** Kelly C. Chang, Natalia A. Trayanova

**Affiliations:** 1 Johns Hopkins University, Department of Biomedical Engineering, Baltimore, MD, 21218, USA

## Abstract

The occurrence of atrial fibrillation (AF) is associated with progressive changes in the calcium handling system of atrial myocytes. Calcium cycling instability has been implicated as an underlying mechanism of electrical alternans observed in patients who experience AF. However, the extent to which calcium-induced alternation of electrical activity in the atria contributes to arrhythmogenesis is unknown. In this study, we investigated the effects of calcium-driven alternans (CDA) on arrhythmia susceptibility in a biophysically detailed, 3D computer model of the human atria representing electrical and structural remodeling secondary to chronic AF. We found that elevated propensity to CDA rendered the atria vulnerable to ectopy-induced arrhythmia. It also increased the complexity and persistence of arrhythmias induced by fast pacing, with unstable scroll waves meandering and frequently breaking up to produce multiple wavelets. Our results suggest that calcium-induced electrical instability may increase arrhythmia vulnerability and promote increasing disorganization of arrhythmias in the chronic AF-remodeled atria, thus playing an important role in the progression of the disease.

Atrial fibrillation (AF) is currently the most common sustained arrhythmia and is associated with significant morbidity and mortality, constituting a major public health concern^1^. Advances in the treatment and prevention of AF are needed to address its rising global prevalence and incidence in the coming decades. Therapies which directly target the mechanistic basis of AF in individual patients are currently lacking^2^,^3^. Progress in AF therapy will depend upon advances in our understanding of the various mechanisms that underlie atrial arrhythmogenesis.

Decreased wavelength in the atria is a well-established mechanism of AF that supports conditions for reentry and thereby promotes AF initiation and maintenance^4^. Recently, efforts to understand the mechanisms of AF have focused increasingly on other aspects of AF remodeling, such as calcium handling abnormalities^5^ and alternans^6^, the beat-to-beat alternation in cardiac electrical signals. Alternans is observed in the ventricles of heart failure patients and has been linked to increased arrhythmia susceptibility^7^,^8^. Clinical evidence suggests that atrial alternans may play an analogous role in AF^6^. Narayan *et al*.^9^ observed action potential duration (APD) alternans in the atria of cardioverted AF patients and found that APD alternans began at slower pacing rates (100–180 bpm) as compared to controls. Furthermore, alternans was always observed before AF was induced at fast pacing rates (240–300 bpm). Thus, the authors hypothesized that alternans may be an important clinical marker for AF risk. However, whether alternans observed in AF patients played a significant role in arrhythmogenesis, and whether the underlying cause of alternans might present a promising therapeutic target, is currently unknown.

We recently demonstrated that calcium handling abnormalities occurring secondary to AF remodeling could underlie the APD alternans observed during atrial pacing of AF-cardioverted patients^10^. The goal of the present study was to investigate the arrhythmogenic consequences of calcium-driven APD alternans (CDA) in a realistic 3D computer model of the human atria. We demonstrate that elevated CDA propensity leads to increased arrhythmia vulnerability, complexity, and persistence due to increased repolarization heterogeneity and wavebreak. The results of this study provide insight into the arrhythmogenic effects of CDA secondary to AF-induced electrical remodeling and suggest that alternans may have clinical significance as a risk stratifier and therapeutic target in AF patients.

## Results

### Arrhythmia vulnerability following an S1–S2 induction protocol

**Figure 1:**
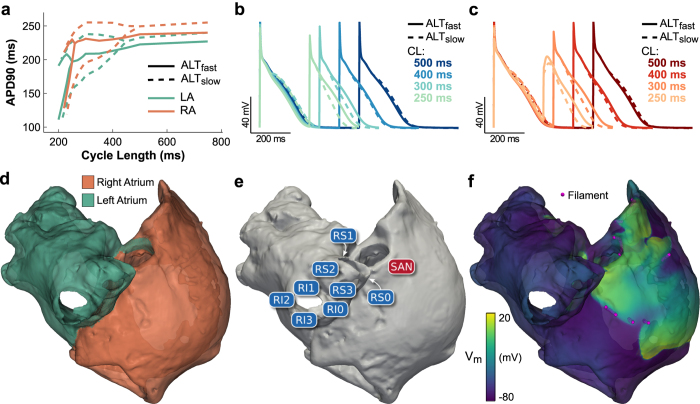
Human atria model. (**a**) Single cell APD restitution for ALT_fast_ (solid line) and ALT_slow_ (dashed line) AP models, using left atrium (LA, green) or right atrium (RA, orange) chronic AF K^+^ current densities from Grandi *et al*.^45^ Bifurcation of the APD restitution curve indicates alternans occurring at that pacing CL. (**b**) LA cell APs for the ALT_fast_ and ALT_slow_ models at different pacing CLs. (**c**) RA cell APs for the ALT_fast_ and ALT_slow_ models at different pacing CLs. (**d**) LA and RA regions in the human atria model. (**e**) Stimulus locations. (**f**) Detection of filaments during arrhythmia.

We examined the effect of increased propensity to CDA on arrhythmia vulnerability in 3D models of the human atria incorporating both electrical (shortened AP) and structural (decreased conductivity) remodeling associated with AF. Two human atrial action potential models were used to simulate membrane kinetics: ALT_fast_, with single-cell alternans occurring only at fast pacing rates (cycle length (CL) ≤250 ms, see Fig. 1a–c and Fig. S1, solid lines); and ALT_slow_, with single-cell alternans occurring at slower pacing rates (CL ≤ 400 ms, see Fig. 1a–c and Fig. S1, dashed lines). Increased alternans propensity in the ALT_slow_ cell model resulted from reduced ryanodine receptor (RyR2) inactivation, the detailed mechanisms of which were explored in our previous simulation study^10^.

**Figure 2:**
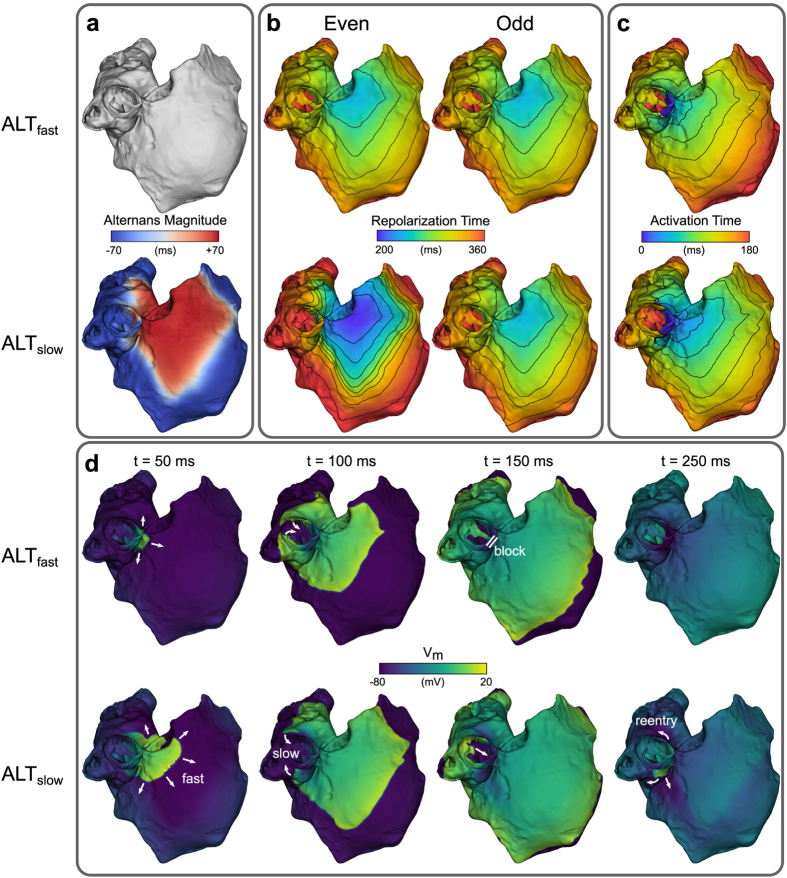
Discordant APD alternans during S1 pacing and induction of reentry by an S2 stimulus. (**a**) APD alternans magnitude. Regions of opposite phase are shown in different colors (red vs. blue). (**b**) Time to 90% repolarization after even (left) and odd (right) paced beats. (**c**) Activation time maps after an S2 stimulus was delivered from RS0 (see Fig. 1E) at a coupling interval of 280 ms following an even S1 beat. (**d**) Transmembrane potential (V_m_) maps showing conduction block and reentry following the S2 stimulus in panel (**c**). Top rows: ALT_fast_. Bottom rows: ALT_slow_.

To induce arrhythmia, an S1–S2 pacing protocol was performed in atria incorporating either ALT_fast_ or ALT_slow_ membrane kinetics. During S1 pacing at 300-ms CL, large-magnitude APD alternans was evident in ALT_slow_ but was absent in ALT_fast_ (Fig. 2a). Furthermore, APD alternans in ALT_slow_ was spatially discordant (Fig. 2a, bottom, red and blue areas out of phase), due to its interaction with conduction velocity (CV) restitution^11^. Although CV restitution slope was virtually identical in ALT_fast_ and ALT_slow_ simulations (Fig. S2a), discordance occurred during S1 pacing in ALT_slow_ but not ALT_fast_ because alternation of the diastolic interval in ALT_slow_ (Fig. S2b) engaged different regions of the CV restitution curve. Accordingly, repolarization times not only varied from beat to beat, but also varied spatially within a single beat (Fig. 2b, bottom). In particular, the even beat in the ALT_slow_ model displayed the most spatial heterogeneity, with areas of early (purple) and late (red) repolarization separated by closely spaced isochrones.

When an S2 beat was delivered to the right superior pulmonary vein (RSPV) at a coupling interval (CI) of 280 ms after the even beat, refractory tissue around the pulmonary vein blocked conduction towards the left atrium (LA) in both ALT_fast_ and ALT_slow_ models (Fig. 2c,d). The wavefronts propagated first into the right atrium (RA) and eventually back around the RSPV into the LA (Fig. 2c). In ALT_fast_, propagation around the RSPV was fast, resulting in the wavefront colliding with the refractory tail of the S2 wave and terminating the reentrant activity (Fig. 2d, top, and Supplementary Video S1). In ALT_slow_, however, differences in the diastolic interval in areas of early and in those of late repolarization led to differences in CV as a result of steep CV restitution (Fig. S2a). Propagation towards the RA was fast while propagation around the RSPV was slow (Fig. 2b), resulting in the wavefront reaching the RSPV later than in ALT_fast_, after refractory tissue from the S2 wave had already recovered. Thus, reentry was able to continue around the RSPV for several cycles (Fig. 2d, bottom, and Supplementary Video S2).

**Figure 3:**
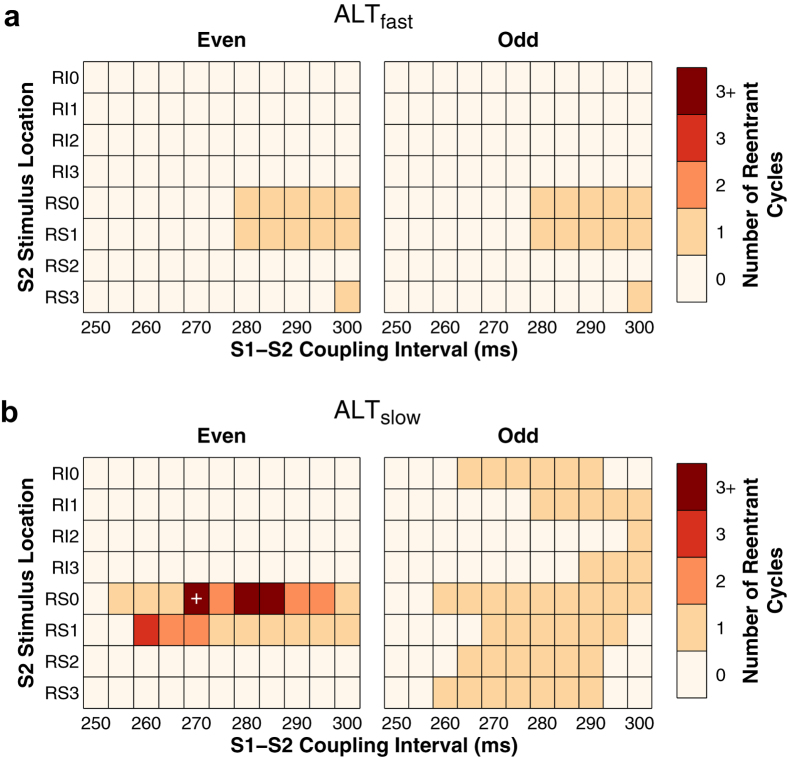
Vulnerability of atria to reentry. Summary of results for ALT_fast_ (**a**) and ALT_slow_ (**b**) simulations. The duration of post-S2 arrhythmia was quantified by the number of times that reentry propagated around the pulmonary veins (referred to as a reentrant cycle). The period of reentries that sustained beyond one reentrant cycle was approximately 250 ms. When post-S2 reentrant activity lasted longer than three reentrant cycles, arrhythmia broke up into multiple wavelets (labeled “3+”). The simulation indicated by the white cross had arrhythmia lasting longer than 2 s.

The S1–S2 protocol was repeated for eight different stimuli locations in the right pulmonary veins, at 11 different CIs following an even or odd S1 beat, for both the ALT_fast_ and ALT_slow_ models (352 atrial simulations total, Fig. 3). As expected, outcomes for ALT_fast_ simulations were identical following even and odd beats, since repolarization patterns did not change from beat to beat (Figs 3a and 2b). Most ectopic beat locations and CIs did not produce reentrant activity in ALT_fast_. Ectopic beats at only three locations (RS0, RS1, and RS3) resulted in post-S2 reentry over a narrow range of CIs, which did not sustain after reentry propagated once around the pulmonary vein (referred to as one reentrant cycle).

In contrast, all eight ectopic beat locations produced post-S2 reentrant activity in ALT_slow_ (Fig. 3b). After the odd beat, any post-S2 activity in ALT_slow_ terminated after one reentrant cycle, as in the ALT_fast_ simulations. After the even beat, however, >1 reentrant cycles were induced at two S2 stimulus locations in ALT_slow_ (RS0 and RS1). These stimuli were located adjacent to areas of early repolarization during the previous S1 beat (purple areas in Fig. 2b, bottom left). Because of the steep repolarization gradients during the even S1 beat, stimuli such as RS3, which experienced similar repolarization times but were not adjacent to areas of early repolarization, had dramatically different outcomes. With RS3, the S2 stimulus was surrounded by refractory tissue at all CIs and resulted in propagation failure.

In some cases, reentry around the pulmonary vein degenerated into fibrillation-like conduction. At RS0, three CIs resulted in reentry that broke up into multiple wavelets after about three cycles. In one simulation (RS0, CI = 270 ms), arrhythmia sustained >2 s after the S2 stimulus, during which the initial reentry around the RSPV eventually terminated and an unstable scroll wave later appeared and meandered in the RA. These results demonstrate that repolarization heterogeneity induced by CDA resulted in increased atrial vulnerability to arrhythmia induction by ectopic beats.

### Arrhythmia complexity during fast pacing

**Figure 4:**
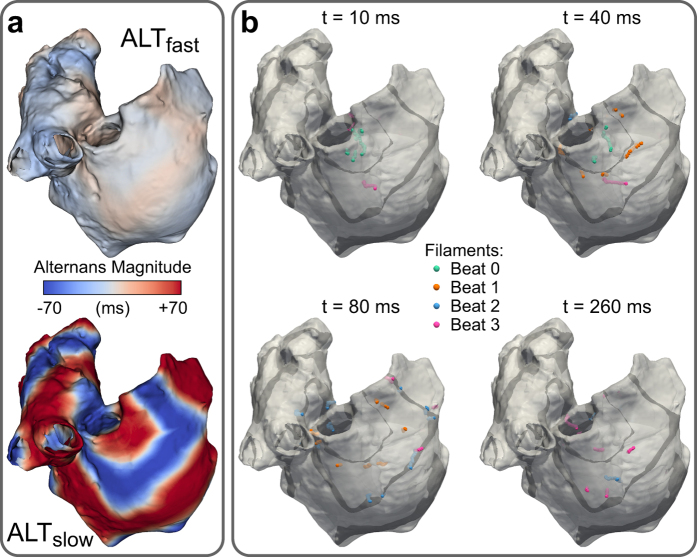
Discordant APD alternans and filaments during fast pacing. (**a**) APD alternans magnitude in ALT_fast_ (top) and ALT_slow_ (bottom) during pacing at 270-ms CL. Regions of opposite phase are shown in different colors (red vs. blue). (**b**) Nodal surfaces (black) and filaments (color) in ALT_slow_ during 260-ms CL pacing. Snapshots show filament locations at different times after a paced beat from SAN region. Filaments from the first four beats are shown in different colors on the same snapshot for comparison.

**Figure 5:**
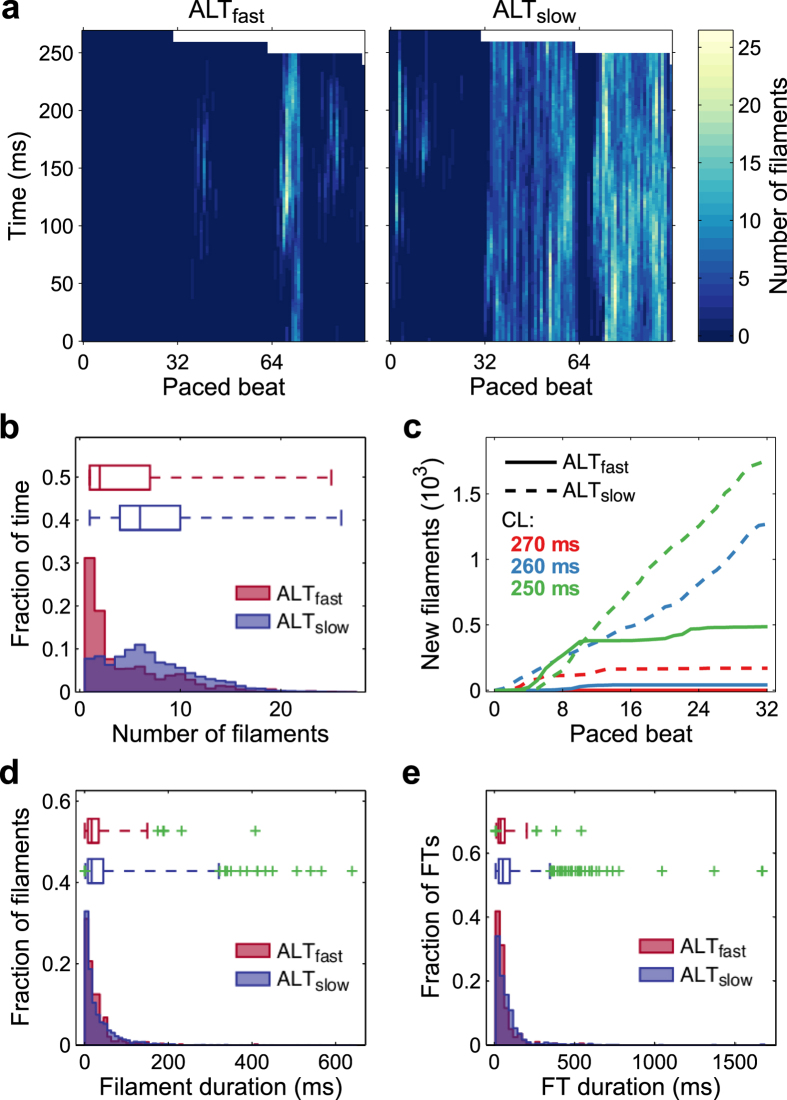
Filament dynamics during fast pacing. (**a**) Heatmaps of the number of distinct filaments present over time in ALT_fast_ (left) and ALT_slow_ (right). (**b**,**d**,**e**): Histograms and adjusted box-whisker plots for ALT_fast_ (red) and ALT_slow_ (blue). Green crosses indicate outliers. (**b**) Number of filaments during arrhythmia. Fraction of time indicates the number of time points during which a particular number of filaments was present, normalized by the total number of time points during which any number of filaments greater than zero was present. (**c**) Cumulative number of new filaments formed in ALT_fast_ (solid line) and ALT_slow_ (dotted line) at pacing CLs of 270 ms (red), 260 ms (blue), and 250 ms (green). (**d**) Filament duration. (**e**) Filament tree (FT) duration.

We next investigated the effect of CDA on arrhythmia complexity in the ALT_fast_ and ALT_slow_ atria models in a new set of simulations in which wave breakup and reentry were induced during a dynamic pacing protocol. The atria were paced from the SAN region at progressively faster rates (from 750-ms to 250-ms CL, 32 cycles at each pacing rate, see Methods). Complexity was quantified by tracking filaments, which are the organizing centers of scroll waves where phase singularities (PSs) occur. Discordant APD alternans developed at 270- and 350-ms CL pacing in ALT_fast_ and ALT_slow_, respectively. During 270-ms CL pacing, several discordant regions were present in both models, but APD alternans magnitude was noticeably larger in ALT_slow_ (red and blue regions, Fig. 4a). As pacing rate increased to 260-ms CL, this repolarization heterogeneity in ALT_slow_ led to wavebreak and filament formation near nodal lines, where regions with alternans of opposite polarity meet (Fig. 4b and Supplementary Video S3). Filaments appeared earlier (during 270-ms CL pacing) and were present longer in the ALT_slow_ model (Fig. 5a). During arrhythmia (i.e. at time points when filaments were present), ALT_slow_ tended to have significantly more filaments than ALT_fast_ (median was 2 and 6 filaments for ALT_fast_ and ALT_slow_, respectively), indicating that CDA increased the complexity of arrhythmia (Fig. 5b and Table 1).

We investigated two possible contributors to the increased number of filaments in ALT_slow_: additional new filament formation or increased filament duration. Figure 5c compares new filament formation during fast pacing (270-ms to 250-ms CL) in ALT_fast_ and ALT_slow_. New filaments appeared steadily during 260-ms and 250-ms CL pacing in ALT_slow_ but slowed in ALT_fast_ after about 10 beats. Thus, to a large degree, new filament formation accounted for the increased number of filaments present in ALT_slow_.

We also compared differences in individual filament persistence during fast pacing. The total number of filaments during this pacing period was 527 and 3197 for ALT_fast_ and ALT_slow_, respectively. Short-lived filaments dominated both ALT_fast_ and ALT_slow_ filament duration distributions (median duration was 17 ms for both, Fig. 5d), and the difference in filament durations was not significant (Table 1). However, the variance in filament durations of the ALT_fast_ versus ALT_slow_ simulations was significantly different (1247.7 vs. 2432.6, *AB* = 525287.5, *p* = 0.003), with ALT_slow_ exhibiting an increased proportion of long-duration filaments (Fig. 5d, top). Furthermore, 9 of the 14 long-lived filament outliers in the ALT_slow_ simulation persisted longer than 400 ms, whereas only 1 of the 6 long-lived outliers did so in the ALT_fast_ simulation. Thus, long lasting filaments occurred more frequently during fast pacing in atria with increased propensity to CDA.

Regional differences in the density of points at which PSs occurred over time were observed during fast pacing. In ALT_fast_, there was higher phase singularity density in the LA than in the RA (Supplementary Fig. S3). In ALT_slow_, phase singularity density was higher in the RA than in the LA except during 250-ms CL pacing, when the reverse was true. This was perhaps due to shorter APD in the LA (Fig. 1a), which allowed the LA to support more scroll waves.

### Arrhythmia persistence during fast pacing

New filaments can form when wavebreak creates new scroll waves, or when preexisting scroll waves interact or break up, resulting in the bifurcation and/or amalgamation of filaments. The proportion of filaments that formed from preexisting filaments during fast pacing (270-ms to 250-ms CL) was higher for ALT_slow_ (0.57 vs. 0.51, 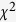 = 6.02, *p* = 0.014). Likewise, the proportion of filaments that were involved in forming new filaments was also higher for ALT_slow_ (0.55 vs. 0.50, 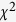 = 3.87, *p* = 0.049). This suggested that increased filament interaction contributed to arrhythmia maintenance in ALT_slow_.

To further characterize the dynamics of filament formation and persistence, we analyzed filament trees (FTs), defined as the set of filaments that interact with each other spatiotemporally. During fast pacing, there were 198 and 976 total FTs in ALT_fast_ and ALT_slow_, respectively. In both simulations, most FTs were short-lived (median duration was 37.5 ms and 52 ms for ALT_fast_ and ALT_slow_, respectively) (Fig. 5e), but FT duration tended to be longer in ALT_slow_ (Table 1). The distribution of FT durations in ALT_slow_ was more right-skewed, with many more long-duration FT outliers (Fig. 5e, top).

**Figure 6:**
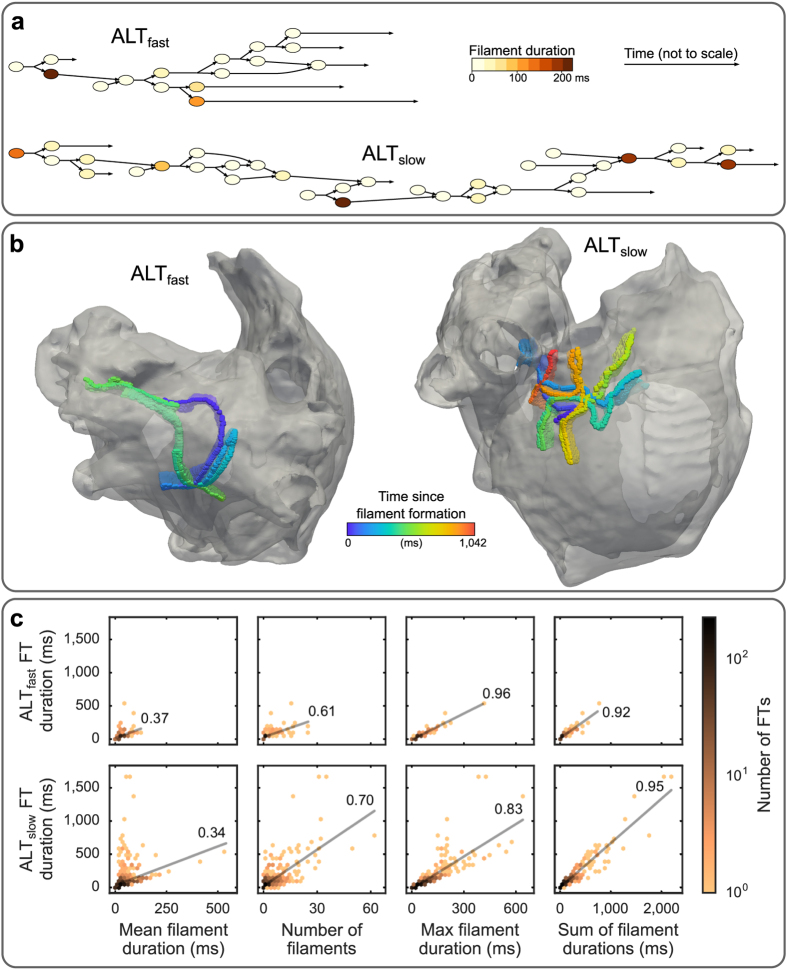
Composition of FTs during fast pacing. (**a**) Graphs of FT branching structure from two example FTs in ALT_fast_ (top) and ALT_slow_ (bottom) simulations. Filament duration is indicated by node colors; time is not drawn to scale. (**b**) Trajectories of ALT_fast_ (left) and ALT_slow_ (right) FTs from panel (**a)**. (**c**) Correlation between FT duration and FT composition metrics. Top: ALT_fast_. Bottom: ALT_slow_. Left to right: mean filament duration, number of filaments, maximum filament duration, and sum of filament durations. Linear best fit lines and Pearson correlation coefficients are shown in each plot.

Two examples of long-lived FT outliers from ALT_fast_ and ALT_slow_ are presented in Fig. 6a,b and Supplementary Videos S4 and S5. Although the temporal branching patterns of FTs could be quite complex, several FTs displayed an overall linear structure (Fig. 6a), indicating that each FT was dominated by a single scroll wave. The lifetimes of these scroll waves were often punctuated by wavebreak, resulting in the branching of a single filament into several short-lived filaments (<25 ms). The ALT_fast_ scroll wave meandered around the left atrial roof and lasted 540 ms (Fig. 6b, left). During its lifetime, one wavebreak occurred, separating the FT into two long-duration filaments (>100 ms) occurring before and after the wavebreak (Fig. 6a, top). The ALT_slow_ scroll wave meandered in a limited region on the right atrial wall near the superior vena cava and lasted 1,043 ms, substantially longer than the ALT_fast_ FT (Fig. 6b, right). Four wavebreak episodes occurred, separating the ALT_slow_ FT into five long-duration filaments (>75 ms, Fig. 6a, bottom). Thus, these patterns suggested that wavebreak contributed to increased persistence of scroll waves in ALT_slow_ as compared to ALT_fast_.

In order to evaluate how representative these FTs were of general differences in FT composition between ALT_fast_ and ALT_slow_, we assessed four different FT composition metrics: mean filament duration, number of filaments, maximum filament duration, and sum of filament durations. The averages of each of these metrics over all FTs within a simulation, and statistical tests of the differences between simulations, are reported in Table 1. For all FT composition metrics, ALT_slow_ tended to have larger values than ALT_fast_, with statistical significance in all metrics except mean filament duration. Not surprisingly, these metrics were also positively correlated with longer FT duration (Table 2).

Figure 6c illustrates the relationship between FT duration and FT composition metrics. Although a modest linear trend was present in the relationship between FT duration and mean filament length, FTs with the longest duration deviated significantly from this trend, having very short mean filament duration (Fig. 6c, column 1). FT duration was more strongly correlated with the total number of filaments per FT (column 2), with the maximum filament duration within each FT (column 3), and with the sum of filament durations within each FT (column 4) (Supplementary Table S2).

These statistical trends were consistent with observations in the two example FTs from Fig. 6a,b. In general, the longest-lived FTs were composed of a few long-duration filaments (>75 ms) preceded and/or followed by the numerous short-duration filaments (<25 ms) that formed during wavebreak. Therefore, mean filament duration was weakly correlated with FT duration, but the number of filaments and the sum of filament durations were strongly correlated with FT duration (Table 2 and Fig. 6c). Additionally, maximum filament duration was more strongly correlated with FT duration in ALT_fast_ than in ALT_slow_ (Δ*r* = 0.13 [0.06, 0.17], Table 2). This was because long-lived FTs in ALT_fast_ tended to be composed of only one or two long-duration filaments, while long-lived FTs in ALT_slow_ tended to be composed of many long-duration filaments, due to the occurrence of several wavebreak episodes in each FTs lifetime. Thus, the above results demonstrate that increased wavebreak, resulting from increased propensity to CDA in ALT_slow_, helped to initiate and sustain arrhythmia in the remodeled atria during fast pacing.

## Discussion

APD alternans has been observed at pacing rates as slow as 100–120 bpm in the atria of cardioverted AF patients undergoing electrophysiological study^6^. In a previous simulation study, we examined the underlying cellular mechanisms driving APD alternans in remodeled atrial myocytes, showing that altered RyR2 kinetics and down regulation of L-type calcium current induced by AF remodeling can lead to CDA at slower pacing rates^10^. Many remodeling changes secondary to AF are profibrillatory, leading to a cycle of worsening AF susceptibility over time (the idea that “AF begets AF”). In particular, shortened APD and decreased CV promote reentry and can stabilize scroll waves to help maintain AF^12^. Since alternans promotes breakup and thus destabilizes scroll waves, the persistence of the arrhythmias in the presence of alternans depends critically on restitution properties^13^. In this study, we sought to explore whether alternans occurring due to the calcium-dependent mechanisms we identified previously ultimately increases or decreases arrhythmia susceptibility.

Our results demonstrate that CDA occurring at slow pacing rates in remodeled atrial myocytes can lead to increased arrhythmia vulnerability, complexity, and maintenance. In a biophysically detailed, anatomically realistic model of the human atria, we found that shortened APD and reduced CV alone were not sufficient to render the atria vulnerable to reentry initiation by an ectopic beat from the pulmonary veins. In contrast, when CDA propensity was increased in the atria via the mechanisms we identified previously^10^, reentry could be induced at two of the right pulmonary vein locations tested. The atria model with increased CDA propensity also displayed more frequent wavebreak at fast pacing rates, resulting in more complex arrhythmia. Frequent wavebreak at fast pacing rates was also associated with longer-duration scroll waves, suggesting that increased wavebreak due to calcium handling instability ultimately increased arrhythmia persistence by producing more scroll waves that helped maintain the arrhythmia. These results suggest that elevated CDA propensity due to AF-induced remodeling of calcium handling may provide a basis for new antiarrhythmic strategies for AF.

Three-dimensional computer models of atrial anatomy and electrophysiology provide a powerful tool for dissecting the mechanisms involved in AF arrhythmogenesis^14^,^15^. Previous computational studies have examined the arrhythmogenic role of decreased wavelength due to shortened APD and/or decreased CV^16^,^17^,^18^. Gong *et al*.^19^ tested the effect of electrically driven alternans on AF initiation in a computer model of paroxysmal AF, in which upregulation of L-type calcium current caused in APD prolongation and steepening of APD restitution slope. However, APD and L-type calcium current are unchanged in paroxysmal AF patients^20^ and are decreased in chronic AF patients^21^, so the clinical relevance of their findings may be limited. The cellular origins of APD alternans (e.g. steep APD restitution slope vs. calcium handling instability) are particularly important because the dynamical instabilities resulting from interaction of APD and CV restitution greatly affect spatial APD heterogeneity and hence arrhythmia vulnerability^11^,^22^. The mechanisms explored in this study stem from a more current understanding of the role of calcium handling abnormalities in human AF^10^ and may thus provide better insight into the pathophysiological mechanisms underlying arrhythmogenesis in patients.

Our results demonstrate that in atria, increased propensity to CDA at slower pacing rates can significantly affect arrhythmia susceptibility. CDA created a vulnerable substrate defined by steep repolarization gradients, which allowed an ectopic beat to induce arrhythmia. This mechanism might explain clinical observations of premature beats initiating AF in patients during slow pacing rates when APD alternans was present^23^. Increased propensity to CDA at slower pacing rates also increased the complexity and persistence of arrhythmias occurring during fast pacing. Arrhythmias were characterized by unstable scroll waves which meandered over large areas and frequently broke up, similar to those observed clinically with ECG imaging^24^. Frequent wavebreak due to underlying calcium instability helped perpetuate arrhythmia in the remodeled atria, supporting the view that abnormal calcium handling may play a significant role in the AF substrate of patients with elevated alternans propensity.

The main mechanisms sustaining AF are much debated, with one of the central controversies being whether AF occurs primarily due to organized reentrant drivers or multiple wavelets^25^. Although several lines of evidence suggest that organized reentrant drivers underlie arrhythmias in many AF patients and may be targeted to effectively terminate arrhythmia^26^, patients with longer-duration continuous AF usually do not respond to ablation therapy^24^. Although exploring AF termination was beyond the scope of this study, we hypothesize that the disorganized, multiple-wavelet arrhythmia occurring due to increased CDA propensity in our model would be more difficult to terminate. Thus, CDA may reflect some of the progressive changes that occur in the underlying substrate during long-standing AF which make standard therapies ineffective. However, in the present study, we modeled fibrotic changes due to AF as a global decrease in tissue conductivity, without taking into account specific patterns of structural heterogeneity that can influence arrhythmia organization^27^,^28^,^29^. Further work is needed to determine how interaction of structural and functional mechanisms affect arrhythmia organization in AF^27^,^30^,^31^, particularly in patients that do not respond to ablation therapy.

Recent efforts to identify mechanisms and potential pharmacological targets in AF have shown that suppressing CDA may have significant antiarrhythmic benefits. Calcium transient alternans and abnormal calcium release events have been observed mouse^32^ and canine^33^ models of AF. Treatment with S107 in mouse^32^ and dantrolene in canine hearts^34^ appeared to stabilize RyR2 and effectively reverse abnormal changes in calcium handling to prevent arrhythmia. Our results suggest that similar mechanisms may be at play in human AF as well, since increased propensity to CDA driven by RyR2 dysfunction led to increased arrhythmia vulnerability, complexity, and persistence in our model. Recent evidence for this comes from experiments in human atrial myocytes of control and AF patients, which identified a link between adenosine signaling, alternans, and calcium handling instability, potentially mediated by PKA regulation of RyR2^35^. However, the mechanisms of RyR2 dysregulation in AF are extremely complex^36^ and controversial^37^. Further work needs to be done to clarify the mechanisms of RyR2 regulation and to explore the antiarrhythmic benefits of RyR2-targeting therapies that address the dual role of calcium as both an initiating trigger and vulnerable substrate in AF^5^.

Although the present study focused on the role of alternans in chronic AF, elevated propensity to APD alternans has been observed in paroxysmal AF patients as well^23^,^38^,^39^. However, the mechanisms underlying alternans may differ, since calcium handling is altered between paroxysmal and chronic AF^20^. In paroxysmal AF, evidence suggests that SK channels^40^, acetylcholine-activated K^+^ current^41^, and late Na^+^ current^42^ may play a more prominent role in alternans propensity and arrhythmogenesis. Given the arrhythmogenic implications of APD alternans presented in this and other studies, these differences in the fundamental mechanisms underlying APD alternans in paroxysmal versus chronic AF underscore the need for further research on the diversity of AF mechanisms^2^, in order to develop better targeted, patient-specific therapies.

## Methods

### Human atria model

We used an anatomically realistic computer model of the human atria to investigate the effect of increased CDA propensity on arrhythmia initiation and maintenance in the atria. Model geometry was based on the Visible Human female dataset^43^, which has been used previously to investigate mechanisms of arrhythmogenesis related to AF^17^. To explore the effects of CDA secondary to AF-induced electrical remodeling on arrhythmogenesis, we tested two atria with chronic AF-induced structural and electrical remodeling. Structural changes were modeled by scaling anisotropic heterogeneous tissue conductivities to produce an average CV of 40 cm/s, consistent with slowed conduction in chronic AF^44^ (Supplementary Table S1). Electrical remodeling was based on a human atrial AP model described by Grandi *et al*., which produced shortened APD as observed in chronic AF^45^. These parameters led to alternans onset at fast pacing rates in the first atria model, ALT_fast_. The second atria model, ALT_slow_, incorporated an additional remodeling feature: reduction of RyR2 inactivation rate by 50%, which we have shown previously results in increased propensity to CDA, matching alternans onset at slower pacing rates observed clinically^10^ (Fig. 1a, see Supplementary Methods). Data on ionic heterogeneity in the atria during AF is limited, so only regional AP heterogeneity between the left (Fig. 1b) and right (Fig. 1c) atrium were included in the atria models (Fig. 1d), as described by Grandi *et al*.^45^.

### Numerical methods

A monodomain formulation was used to represent electrical propagation throughout the human atria model^46^. The monodomain and AP model equations were solved together by a finite element approach using the Cardiac Arrhythmia Research Package (CARP; Cardiosolv, LLC)^47^,^48^. A time step of 20 μs was used for all simulations. The mesh contained 2,198,425 nodes and 3,005,729 elements with a mean edge length of 330 μm.

### Arrhythmia induction protocols

To compare arrhythmia dynamics between ALT_fast_ and ALT_slow_ models, we performed a dynamic pacing protocol similar to one used clinically to assess the presence of alternans and induce AF in patients^23^. All nodes in the model were initialized using steady-state values from a single cell paced at 750-ms CL. The atria were paced from the SAN region at 750-ms CL for 20 beats (Fig. 1e), and then for 32 beats at each subsequent pacing CL: 500-ms to 300-ms CL in 50-ms decrements, then 290-ms to 250-ms CL in 10-ms decrements. Arrhythmias were examined (see Methods section on filament dynamics) during the fastest pacing rates when wavebreak and reentry occurred (CL ≤ 270 ms).

To assess arrhythmia vulnerability due to ectopic beats at slower pacing rates, we performed an S1–S2 pacing protocol using the same initial dynamic pacing protocol described above. After the 16^th^ even or odd beat at 300-ms CL pacing, SAN pacing ceased and an ectopic stimulus (S2) was applied to one of eight locations in pulmonary veins at eleven different CIs, ranging from 250 ms to 300 ms. Figure 1e shows location of the S2 stimuli, in the right superior pulmonary veins (RS0–3) or the right inferior pulmonary veins (RI0-3). Post-S2 activity was simulated for 2 s or until activity terminated, whichever came first.

### Quantification of APD alternans

APD was calculated as the time from maximal upstroke velocity to 90% repolarization of the transmembrane potential (V_m_) from phase II amplitude. Alternans magnitude was quantified as the mean change in APD from beat to beat over the last 10 pairs of beats (11 beats total) at a given pacing CL^23^.

### Filament dynamics

Phase singularities are points where the phase of the AP is undefined, occurring at locations of wavebreak or at the tip of a reentrant spiral wave. In 3 D, phase singularities form the vortex filaments of reentrant scroll waves. To robustly detect filaments in our simulations despite variations in AP morphology^49^, we developed a novel method for filament localization, which involved constructing phase maps from activation times (see Supplementary Methods). To gain insight into filament dynamics, we tracked at 1-kHz resolution each filament’s birth, death, and interactions with other filaments, splitting (bifurcation) or merging (amalgamation) to form new filaments. A FT was defined as the set of filaments that interact with each other over time. Spurious filaments associated with brief wavebreak were not included in the dataset to allow interpretation of the temporal branching structure of filament interactions. An example snapshot of filament localization during arrhythmia is shown in Fig. 1f. Details on filament detection can be found in Supplementary Methods.

### Statistical analysis

Adjusted box-whisker plots were used to compare skewed distributions and detect outliers^50^. Chi-squared tests of independence without correction were used to assess differences in filament interaction^51^. The Mann-Whitney *U* statistic was used to test for differences in distribution location. The Ansari-Bradley statistic (*AB*) was used to test for differences in the scale (i.e. variance) of distributions with identical medians^52^. Two-tailed tests were performed with *p* < 0.05 considered significant. Pearson’s correlation coefficient *r* was used to quantify the linear dependence between variables. Significance of Pearson correlations and their differences (Δ*r*) was assessed using a percentile bootstrap method to estimate 95% confidence intervals (Wilcox-Muska confidence intervals) in the independent case, and using the HC4 method in the overlapping dependent case^53^. These methods have been shown to perform well under conditions of nonnormality and heteroscedasticity^54^.

## Additional Information

**How to cite this article**: Chang, K. C. and Trayanova, N. A. Mechanisms of arrhythmogenesis related to calcium-driven alternans in a model of human atrial fibrillation. *Sci. Rep.* **6**, 36395; doi: 10.1038/srep36395 (2016).

**Publisher’s note:** Springer Nature remains neutral with regard to jurisdictional claims in published maps and institutional affiliations.

## Supplementary Material

Supplementary Information

Supplementary Video S1

Supplementary Video S2

Supplementary Video S3

Supplementary Video S4

Supplementary Video S5

## Figures and Tables

**Table 1:** Filament and FT metrics during fast pacing (270-ms to 250-ms CL).

	ALT_fast_	ALT_slow_	*U*	*p*
Duration of arrhythmia (ms)	3328	15894	—	—
Number of filaments during arrhythmia	4.50	7.07	16111484.5	<10^−6^
Number of filaments	527	3197	—	—
Filament duration (ms)	28.4	35.2	808583	0.14
Number of FTs	198	976	—	—
FT duration (ms)	56.0	84.8	82880.5	0.002
**FT composition metrics**				
Number of filaments/FT	2.66	3.28	84377.5	<0.001
Mean filament duration/FT (ms)	33.8	40.8	92282.5	0.32
Max filament duration/FT (ms)	47.8	63.3	84714	0.006
Sum of filament durations/FT (ms)	75.6	115.3	82306.5	<0.001

Mean values for the ALT_fast_ and ALT_slow_ simulations are reported for all metrics except for duration of arrhythmia, number of filaments, and number of FTs. Differences in distribution between the ALT_fast_ and ALT_slow_ simulations were assessed with the Mann-Whitney *U* statistic, with *p* < 0.05 considered significant.

**Table 2:** Pearson correlations between FT duration and FT composition metrics for the ALT_fast_ and ALT_slow_ simulations and the difference in ALT_fast_ and ALT_slow_ correlations (Δ*r*) during fast pacing (270-ms to 250-ms CL).

	ALT_fast_	ALT_slow_	Δ*r*
Number of Filaments	0.61 [0.48 0.74]	0.70 [0.64 0.75]	−0.08 [−0.22 0.05]
Mean Filament Duration	0.37 [0.27 0.51]	0.34 [0.29 0.43]	0.03 [−0.11 0.18]
Maximum Filament Duration	0.96 [0.92 0.98]	0.83 [0.80 0.89]	0.13 [0.06 0.17]
Sum of Filament Durations	0.92 [0.87 0.96]	0.95 [0.93 0.96]	−0.03 [−0.08 0.02]

Confidence intervals at 95% shown in brackets.
