# Supplementary material for: Mechanisms of arrhythmogenesis related to calcium-driven alternans in a model of human atrial fibrillation
Source: Sci Rep. 2016 Nov 4;6:36395. doi: 10.1038/srep36395 (PMC5095679; doi:10.1038/srep36395)
Supplement: Supplementary Information [file srep36395-s1.pdf]

MECHANISMS OF ARRHYTHMOGENESIS RELATED TO CALCIUM-DRIVEN ALTERNANS IN A MODEL OF HUMAN ATRIAL FIBRILLATION

Kelly C. Chang and Natalia A. Trayanova

Johns Hopkins University, Department of Biomedical Engineering, Baltimore, MD, 21218, U.S.A.

Supplementary Methods

Human atria model

We previously investigated the cellular mechanisms of calcium-driven alternans (CDA) in the Grandi-Pandit-Voigt human atrial action potential model[1]. Our findings demonstrated that changes in the ryanodine receptor inactivation rate (ki_{Ca}) could modulate the propensity of chronic atrial fibrillation (cAF)-remodeled cells to CDA. When ki_{Ca} was decreased by 50% in the cAF model described by Grandi *et al.*[2], alternans onset cycle length (CL), mean action potential duration (APD) at onset, and APD alternans magnitude at onset agreed well with data obtained during atrial pacing in cardioverted patients with persistent atrial fibrillation (AF). The default cAF model described by Grandi *et al.* and the cAF model with 50% ki_{Ca} were used to compare the effects of altered CDA propensity on arrhythmogenesis in the human atria model.

The ionic models were modified for use in tissue simulations by replacing fast sodium current kinetics with that of the LRd model[3], as done previously[1]. Rule-based fiber orientation was included in the human atria model to produce realistic activation patterns[4]. Anisotropic conductivity ratios varied regionally as specified by Krueger *et al.*[4], with conductivities scaled uniformly to produce slow conduction consistent with measurements in AF patients[5] (Supplementary Table S1).

Filament calculations and filtering

We developed an algorithm to find filaments using activation time data. When transmembrane potential (V_m) of a particular node, n_i, crossed above the activation threshold (-40 mV), an activation time value (ATV) of 1 was assigned to n_i for that time point. Any activations occurring within 50 ms of a previous activation at the same node were discarded. To generate smooth activation wavefronts, if any adjacent node (sharing the same element) activated within the 20 ms after the activation of n_i, then n_i was assigned an ATV of 1 from the time of its own activation up to and including the time of the last activation of an adjacent node. For the subsequent 20 ms, the ATV at n_i was decremented by 0.05/ms. Node n_i was assigned an ATV of 0 at all other times. Phase singularities were located as described by Iyer and Gray[6]. The phase of n_i (θ_{n_i}) at each time point (t) was computed by the following function:

$$\theta_{n_i}(t) = \text{arctan2}\left(\text{ATV}_{n_i}(t+\tau) - \text{ATV}^*, \text{ATV}_{n_i}(t) - \text{ATV}^*\right)$$

where τ = 1 ms and ATV^* = 0.5. The choice of these parameters followed naturally from the prescribed time course of the ATV variable after activation. Phase singularities (PSs) were identified by calculating the line integral of the phase around every face in the model[7]. All nodes of any face containing a PS (line integral = $\pm 2\pi$) were assigned as being associated with a PS. A filament was identified as the set of adjacent nodes associated with PSs.

Filaments were tracked spatiotemporally at a resolution of 1 kHz, as described by Clayton and Holden[8]. If only one filament from the current time step shared nodes with or was adjacent to exactly one filament from the previous time step, it was considered the same filament. However, when a filament shared nodes with or was adjacent to more than one filament from the previous time step, the filament in the current time step was considered a new filament generated from the merging of the filaments of the previous time step. Conversely, when multiple filaments shared

nodes with or were adjacent to a filament from the previous time step, the filaments in the current time step were considered new filaments generated from the splitting of the filament in the previous time step. A filament tree (FT) was defined as the set of filaments that interacted over time.

To simplify analysis of filament populations, we removed spurious filaments, defined as any filament lasting <6 ms which did not arise from previous filaments and did not generate new filaments. At the beginning or end of a FT, these were associated with complex conduction block patterns that were not of interest in this study. In the middle of a FT, these were often associated with minute wavebreaks. When multiple filaments merged into one, or one filament split into multiple, spurious filaments were removed from the set of multiple filaments such that the longest-lived filament (whether spurious or not) always remained. In the event that at this branching point, all but one filament was removed, the filament before and after this point would be considered the same filament (i.e. the branching point disappeared).

References

1. Chang, K. C., Bayer, J. D. & Trayanova, N. A. Disrupted Calcium Release as a Mechanism for Atrial Alternans Associated with Human Atrial Fibrillation. *PLoS Comput. Biol.* **10,** e1004011 (2014).
2. Grandi, E. *et al.* Human atrial action potential and Ca2+ model: sinus rhythm and chronic atrial fibrillation. *Circ. Res.* **109,** 1055–66 (2011).
3. Luo, C. H. & Rudy, Y. A dynamic model of the cardiac ventricular action potential. I. Simulations of ionic currents and concentration changes. *Circ. Res.* **74,** 1071–96 (1994).
4. Krueger, M. W. *et al.* in *Functional Imaging and Modeling of the Heart* (eds. Metaxas, D. N. & Axel, L.) **6666,** 223–232 (Springer Berlin Heidelberg, 2011).
5. Konings, K. T. *et al.* High-density mapping of electrically induced atrial fibrillation in humans. *Circulation* **89,** 1665–80 (1994).
6. Iyer, A. N. & Gray, R. A. An Experimentalist's Approach to Accurate Localization of Phase Singularities during Reentry. *Ann. Biomed. Eng.* **29,** 47–59 (2001).
7. Larson, C., Dragnev, L. & Trayanova, N. Analysis of Electrically Induced Reentrant Circuits in a Sheet of Myocardium. *Ann. Biomed. Eng.* **31,** 768–780 (2003).
8. Clayton, R. H. & Holden, A. V. A method to quantify the dynamics and complexity of re-entry in computational models of ventricular fibrillation. *Phys. Med. Biol.* **47,** 225–238 (2002).

Supplementary Tables

Supplementary Table S1: Human atria model regional conductivities

Region	Conductivity	Krueger et al. 2011 value (S/m)	Model Value (S/m)	g_l/g_t	Conduction Velocity (m/s)
Myocardium, appendages	g_t	0.07500	0.04748	3.83	0.27
	g_l	0.28125	0.18188		0.66
Valve rings	g_t	0.07500	0.04748	3.71	0.27
	g_l	0.27225	0.17622		0.64
Isthmus	g_t, g_l	0.07500	0.04748	1.00	0.27
Bachmann's bundle	g_t	0.18100	0.11489	3.97	0.50
	g_l	0.70228	0.45584		1.10
Crista terminalis	g_t	0.07500	0.04748	6.68	0.27
	g_l	0.49200	0.31730		0.90
Pectinate muscles	g_t	0.03000	0.01905	23.77	0
	g_l	0.69750	0.45284		1.10
Sinoatrial node	g_t, g_l	0.27500	0.17796	1.00	0.65

Supplementary Table S2: Comparison of FT composition correlations

Pearson correlation between FT duration and mean filament duration was compared to the correlation between FT duration and other FT composition metrics for the ALT_{fast} and ALT_{slow} simulations during fast pacing (cycle lengths of 270 ms to 250 ms). Confidence intervals at 95% are shown in brackets.

	ALT_{fast}	ALT_{slow}
**Number of filaments**	0.25 [-0.15 0.64]	0.36 [0.14 0.58]
**Maximum filament duration**	0.59 [0.44 0.74]	0.49 [0.37 0.62]
**Sum of filament durations**	0.55 [0.24 0.86]	0.61 [0.48 0.74]

Supplementary Figures

Supplementary Figure S1: APD alternans in single cell model

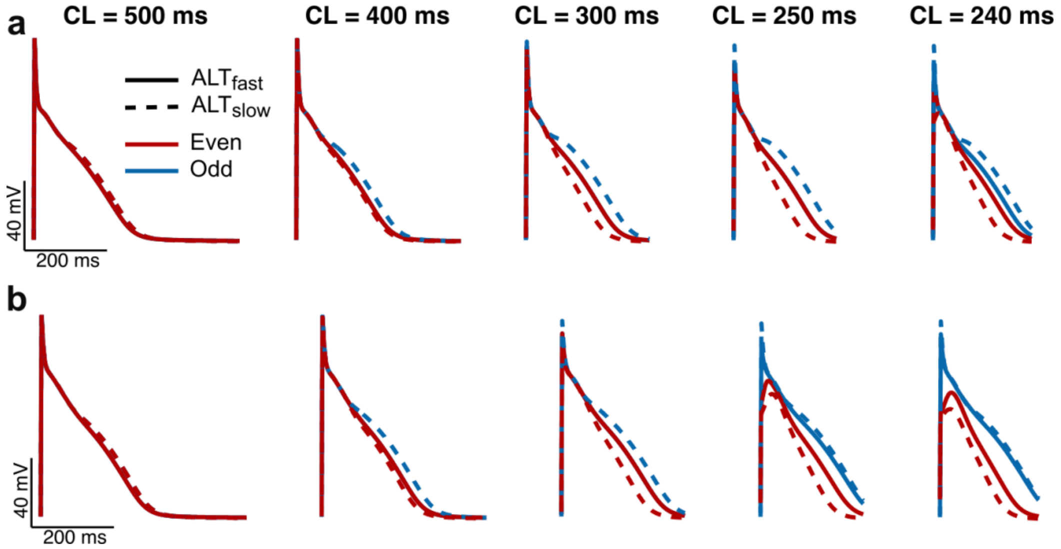

Transmembrane potential in left (**a**) and right (**b**) atrial single cell models. Traces from even (red) and odd (blue) beats are superimposed to show alternans in APD. With ALT_{fast} membrane kinetics, significant APD alternans (>5% of mean APD) was observed in both left and right atrial cells (solid lines) at CL ≤ 400 ms. With ALT_{slow} membrane kinetics (dashed lines), alternans only occurred at CL ≤ 250 ms in right atrial cells and at CL ≤ 240 ms in left atrial cells.

Supplementary Figure S2: CV restitution and APD restitution in tissue strand model

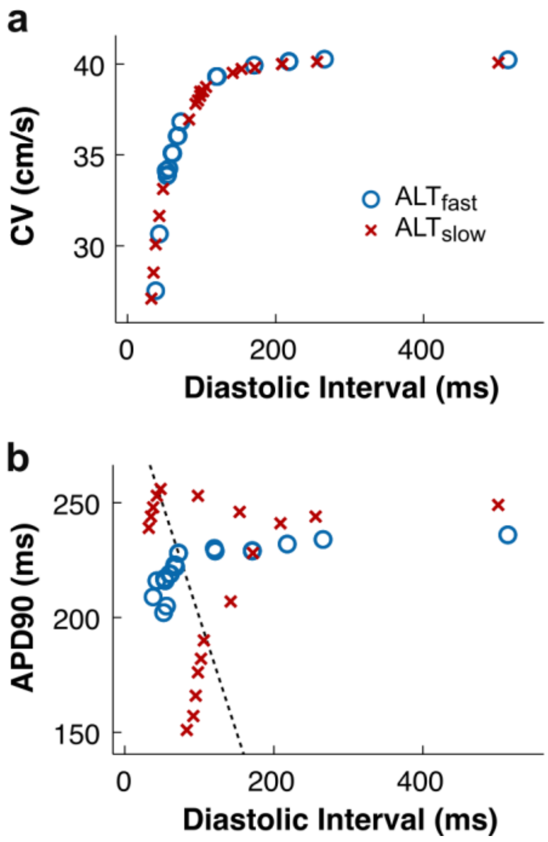

Conduction velocity (CV) restitution (**a**) and APD restitution (**b**) were assessed in a tissue strand model (dimensions 0.33 mm × 0.33 mm × 9.9 mm, 0.33-mm discretization). A dynamic pacing stimulus (see Methods in main text) was applied at one end of the tissue strand. CV was measured between sites located 6.27 mm and 8.58 mm away from the pacing site. APD was measured at a site 6.6 mm away from the pacing site. Black dotted line indicates the APD-diastolic interval relationship at a pacing CL of 300 ms. Alternans in APD occurred with ALT_{slow} membrane kinetics (red Xs) but not ALT_{fast} membrane kinetics (blue circles) at 300-ms CL.

Supplementary Figure S3: Phase singularity density during fast pacing

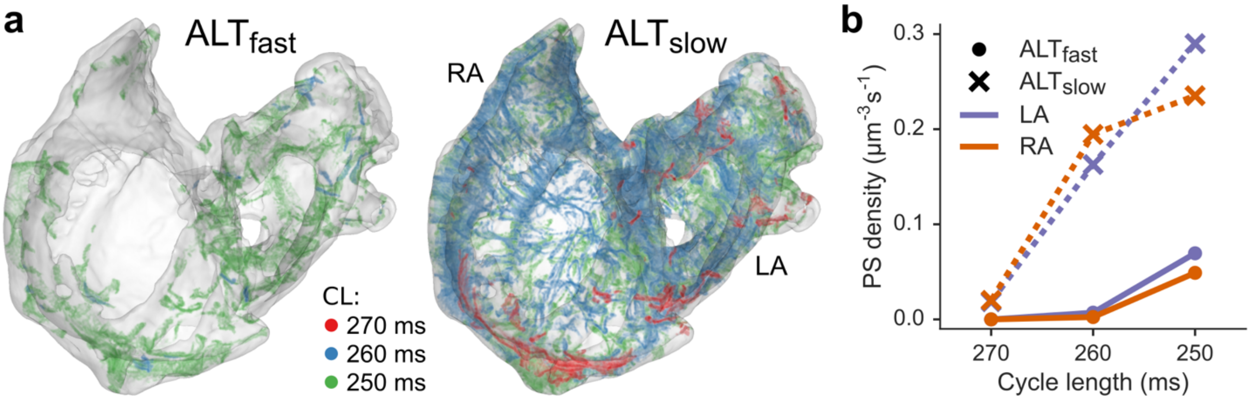

(**a**) Locations of phase singularity (PS) points in ALT_{fast} (left) and ALT_{slow} (right) during pacing at CLs of 270 ms (red), 260 ms (blue), and 250 ms (green). (**b**) Mean density of points at which PSs occurred over time in the LA (purple) and RA (orange) for ALT_{fast} (circles) and ALT_{slow} (x's).

Supplementary Videos

Supplementary Video S1

S2 stimulus delivered at RS0 with a coupling interval of 280 ms following an even S1 beat in ALT_{fast}.

Supplementary Video S2

S2 stimulus delivered at RS0 with a coupling interval of 280 ms following an even S1 beat in ALT_{slow}.

Supplementary Video S3

First five beats during 260-ms CL pacing in ALT_{slow}.

Supplementary Video S4

Example filament tree from ALT_{fast} simulation during fast pacing at 250-ms CL.

Supplementary Video S5

Example filament tree from ALT_{slow} simulation during fast pacing at 260-ms CL.
